# Fibrous scaffolds loaded with BMSC-derived apoptotic vesicles promote wound healing by inducing macrophage polarization

**DOI:** 10.1016/j.gendis.2024.101388

**Published:** 2024-08-09

**Authors:** Xudong Su, Jianye Yang, Zhenghao Xu, Li Wei, Shuhao Yang, Feilong Li, Min Sun, Yingkun Hu, Wenge He, Chen Zhao, Li Chen, Yonghua Yuan, Leilei Qin, Ning Hu

**Affiliations:** a Department of Orthopedics, The First Affiliated Hospital of Chongqing Medical University, Chongqing 400016, China; b Laboratory of Orthopedics, Chongqing Medical University, Chongqing 400016, China; c Department of Knee Joint Sports Injury, Sichuan Provincial Orthopedic Hospital, Chengdu, Sichuan 610042, China; d Research Center for Pharmacodynamic Evaluation Engineering Technology of Chongqing, College of Pharmacy, Chongqing Medical University, Chongqing 400016, China

**Keywords:** Apoptotic body, Extracellular vesicles, Macrophage polarization, Mesenchymal stem cells, miR-21a-5p, Polycaprolactone, Wound healing

## Abstract

Macrophages play a key role in wound healing. Dysfunction of their M0 polarization to M2 leads to disorders of the wound immune microenvironment and chronic inflammation, which affects wound healing. Regulating the polarization of M0 macrophages to M2 macrophages is an effective strategy for treating wound healing. Mesenchymal stem cells (MSCs) deliver endogenous regulatory factors via paracrine extracellular vesicles, which may play a key role in wound healing, and previous studies have shown that apoptotic bodies (ABs) are closely associated with inflammation regression and macrophage polarization. However, the specific regulatory mechanisms involved in ABs remain unknown. In the present study, we designed an MSC-AB (MSC-derived AB)-loaded polycaprolactone (PCL) scaffold, evaluated the macrophage phenotype and skin wound inflammation *in vivo* and *in vitro*, and explored the ability of MSC-AB-loaded PCL scaffolds to promote wound healing. Our data suggest that the PCL scaffold regulates the expression of the CCL-1 gene by targeting the delivery of mmu-miR-21a-5p by local sustained-release MSC-ABs, and drives M0 macrophages to program M2 macrophages to regulate inflammation and angiogenesis, thereby synergistically promoting wound healing. This study provides a promising therapeutic strategy and experimental basis for treating various diseases associated with imbalances in proinflammatory and anti-inflammatory immune responses.

## Introduction

The skin accounts for approximately 16% of the body weight and is the largest organ of the body.^1^ Once the skin barrier is damaged, the body initiates precise regulation of wound contraction, hemostasis, inflammation, angiogenesis, granulation tissue proliferation, and epithelial remodeling to promote wound healing.^2^^,^^3^ Macrophages play a crucial role in the inflammatory response of wound tissue, and their active plasticity allows them to regulate tissue damage and repair functions, while macrophage-mediated inflammatory responses are closely associated with wound healing.^4^ Macrophages polarized by environmental signals can be broadly classified into two main groups: classically activated macrophages with pro-inflammatory properties (M1), whose prototypical activating stimuli are interferon-gamma and lipopolysaccharide, and macrophages with anti-inflammatory and wound healing functions alternative to activation (M2), further subdivided into M2a (after exposure to interleukin (IL)-4 or IL-13), M2b (immune complexes in combination with IL-1β or lipopolysaccharide), and M2c (IL-10, transforming growth factor-beta (TGF-β) or glucocorticoids).^5^^,^^6^ The phenotype of macrophages is influenced by the microenvironment of the wound and evolves during the healing process from a pro-inflammatory (M1) profile in the early stages to a less inflammatory pro-healing (M2) phenotype in the later stages.^7^ M1 macrophages dominate in the early stages of wound healing and display phagocytic activity and the secretion of proinflammatory cytokines such as IL-1β, IL-6, IL-12, tumor necrosis factor-alpha (TNF-α), and oxidative metabolites to remove pathogens, tissue debris, and senescent cells from the wound surface.^8^ In the middle to late stages, the M0 macrophage phenotype is reprogrammed to an anti-inflammatory M2 phenotype, secreting anti-inflammatory cytokines such as IL-4 and IL-10 to suppress the local inflammatory response and producing vascular endothelial growth factor (VEGF) to promote angiogenesis and stabilization.^9^ Dysfunctional M0 macrophage polarization to M2 macrophage polarization, reduced M2 macrophage numbers, and diminished anti-inflammatory and angiogenic capacity are reasons why trauma results in the long-term persistence of nonhealing in the inflammatory phase.^10^ Therefore, effective regulation of the polarization of M0 macrophages to M2-type macrophages, which exert anti-inflammatory effects and promote angiogenesis, will significantly improve wound healing.

Mesenchymal stem cells (MSCs), an important endogenous cellular reservoir for tissue repair and regeneration, can effectively respond to inflammation and regulate macrophage reprogramming.^11^ Currently, the ability of tissue engineering to promote wound healing has been investigated mainly through the secretion of paracrine growth factors, immune factors, chemokines, and extracellular vesicles by MSCs.^12^ Recent studies have shown that extracellular vesicles produced by bone marrow-derived MSCs can contribute to tissue repair by promoting angiogenesis under a variety of pathological conditions, including skin wound healing, acute kidney injury, and myocardial infarction. In addition, they are widely used as drug delivery systems for cardiovascular diseases, neurodegenerative diseases, liver diseases, lung diseases, and kidney diseases.^13^, ^14^, ^15^ Extracellular vesicles can be divided into three subgroups (exosomes, microvesicles, and apoptotic vesicles) and play a role in intercellular communication by transmitting complex signals.^16^ Apoptotic bodies (ABs) are the largest extracellular vesicles, with a diameter of approximately 50–5000 nm, and are rich in DNA, microRNA, mRNA, proteins, and organelles.^17^ After bone marrow-derived MSCs undergo apoptosis, macrophages rapidly respond to apoptotic signals, recognize and take up apoptotic vesicles within a short period, and trigger the polarization of M0 macrophages to the M2 phenotype, while M2 phenotype macrophages further enhance the function of fibroblasts and synergistically promote skin wound healing.^18^ Therefore, the ABs of MSCs may serve as promising candidates for the development of cell-free therapies and provide new strategies for the treatment of cutaneous wounds.

To further achieve the controlled release of key bioinformatic molecules to M0 macrophages within the wound surface and drive the polarization of M0 macrophages to M2 macrophages, the selection of suitable wound dressings to load therapeutic factors is a promising strategy.^19^ Scaffolds serve as a means of restoring the morphology and function of diseased, damaged, and lost tissues by acting as an extracellular matrix for supporting cells and their fate and function.^20^ Various natural and synthetic biopolymers can be used to fabricate such scaffolds, such as natural biomacromolecules, including silk fibroin, collagen, gelatin, chitosan, and hyaluronic acid, and synthetic biopolymers, including polyethylene glycol, polycaprolactone (PCL), polylactic acid-glycolic acid copolymer, and poly l-lactide.^21^ Previous studies have shown that the loading of MSC-derived extracellular vesicles on heparin-modified 10.13039/100018919PCL scaffolds inhibits thrombosis and calcification in the treatment of cardiovascular disease, thereby improving graft patency and enhancing endothelial and vascular smooth muscle regeneration while inducing M1 macrophage polarization to M2c macrophages.^22^ MSC-exosomes loaded on 10.13039/100018919PCL scaffolds modified with S-nitrosoglutathione reduce the expression of proinflammatory genes in treated macrophages and accelerate osteogenic differentiation in bone defects.^23^ In our study, we prepared 10.13039/100018919PCL scaffolds using an electrospinning technique that is thought to better mimic the physical structure of the extracellular matrix as well as the suitable mechanical properties for the delivery of apoptotic vesicles and wound dressings.^24^ To investigate the specific regulatory mechanism of MSC involvement in apoptotic vesicles, we investigated the mechanism of action of MSC-AB-loaded 10.13039/100018919PCL scaffolds in regulating macrophage polarization for wound healing in a mouse wound model, providing an experimental basis and theoretical rationale for the development of new drugs.

## Materials and methods

### Cell culture

Primary MSCs were derived from bone marrow-derived stem cells (BMSCs) harvested from C57BL/6 mice. Bone marrow was obtained from the femurs and tibias of C57BL/6 mice and was washed and filtered to form single-cell suspensions. Primary BMSCs were cultured in Dulbecco's modified Eagle medium (DMEM) (Gibco, Grand Island, NY, USA) supplemented with 10% fetal bovine serum (Gibco) and 1% penicillin/streptomycin (Invitrogen, Carlsbad, CA, USA) at 37 °C in a 5% CO_2_ cell culture incubator. The medium was changed every 2–3 days. The adherent cells were digested with 0.25% trypsin (MP Biomedicals, Irvine, CA, USA) and passaged *in vitro*, and third- and fourth-generation BMSCs were used for subsequent experiments. A mouse macrophage line (RAW264.7) and 293T cells were obtained from the CAS Cell Bank. The NIH-3T3 mouse fibroblasts used for the fibroblast scratch migration experiments were obtained from Procell Life Science & Technology Co., Ltd. (Wuhan, China).

### Isolation and characterization of BMSC-derived ABs

After BMSCs were cultured in serum-free DMEM for 24 h, staurosporine (0.5 μM) (MedChemExpress, NJ, USA) was added for 12 h to induce apoptosis of MSCs. The medium was then collected and centrifuged at 300 *g* for 10 min to remove cells and debris. After two repeated centrifugations, the supernatant was collected and further centrifuged at 3000 *g* for 30 min to concentrate the ABs into pellets, which were then resuspended in 1× phosphate buffer saline solution (PBS) and stored at −80 °C for subsequent experiments. Protein concentrations were measured using the BCA Protein Assay Kit (Beyotime Biotechnology, Shanghai, China). The purified ABs were characterized by western blotting using primary antibodies against caspase-3, CD9, CD63, GAPDH, and cleaved caspase-3 (rabbit mAb, Cell Signaling Technology, Boston, MA, USA). Dynamic light scattering analysis was performed using a Zetasizer Nano ZSE (Malvern Panalytical, Malvern, England, UK). The morphology of the ABs was observed by scanning electron microscopy (Hitachi, TKY, Japan).

### Preparation of PCL scaffolds by electrospinning

One gram of PCL solid particles was dissolved in 10 mL of dichloromethane to form a 10% (w/v) PCL/dichloromethane solution, which was stirred continuously at room temperature for 2 days until the solution became clear and transparent. The electrospun emulsion was drawn into a 10 mL syringe (with a 21G stainless steel flat-tipped dispensing needle) and placed on a microinjection pump (LongerPump, Baoding, China) for electrospinning, which was performed in a fume hood and collected from the holder via a homemade drum collector. The electrostatic spinning parameters were set as follows: injection rate of 1 mL/h, voltage of 15 kV, receiver speed of 150 rpm, and reception distance of 12–15 cm. The resulting fiber material was dried well in a fume hood and left at room temperature.

### Characterization of PCL scaffolds loaded with BMSC-ABs

PCL scaffolds were dried at room temperature for 1 day. All scaffolds were cut into 10 × 10 mm squares, fixed to the sample stage with double-sided carbon conductive adhesive, and examined by field emission scanning electron microscopy (Hitachi) after 40 s of gold spraying under vacuum with the acceleration voltage set to 10 kV. The scaffold diameters were statistically analyzed by ImageJ software (NIH, Bethesda, MD, USA) to characterize the PCL fiber scaffold morphology.

The PCL scaffold material was also cut into 15 mm diameter circles to fit 24-well culture plates. The materials were sterilized using Co irradiation at a radiation dose of 10 kGy. After sterilization, the materials were fixed at the bottom of the 24-well plates, washed three times with PBS, and then incubated in PBS for 24 h. Subsequently, mouse bone marrow MSC ABs were inoculated at 50 μg/mL on the surface of the materials and incubated in a cell culture incubator at 37 °C for 12 h. The scaffold materials were incubated by scanning electron microscopy (Hitachi) to observe the morphology of the loaded BMSC-AB-PCL fibrous scaffold as a means of characterization.

### The release rate of PCL scaffolds loaded with BMSC-ABs

ABs of mouse bone marrow MSCs were inoculated at 50 μg/mL on the surface of PCL fibrous scaffolds and incubated in an incubator at 37 °C for 12 h. An equal amount of suspension was added to PBS and collected every 12 h. After the protein was measured with a BCA protein assay kit, an equal amount of the protein concentration was measured with a BCA protein assay kit (Beyotime Biotechnology), and then the protein was added to the scaffolds until the protein concentration was less than 5 μg/mL.

### Fluorescently labeled BMSC-ABs and cytophagocytosis

According to the manufacturer's protocol, the cytoskeleton green fluorescent dye phalloidin (Thermo Fisher Scientific, Inc., Alexa Fluor 488, Invitrogen, USA) and exosome membrane red labeling dye (1′-dioctadecyl-3,3,3′,3′-tetramethylindole dicarbapenem, DiD) (Thermo Fisher Scientific) were used to label the purified ABs. ABs were incubated in 5 μg/mL DiD staining solution at 37 °C for 30 min, washed with PBS, and centrifuged at 3000 *g* for 30 min twice. The unattached dye was removed using an ultrafiltration tube (300 kDa, Sigma–Aldrich, Saint Louis, MO, USA). RAW264.7 cells were inoculated in 35 mm confocal dishes at a density of 1 × 10^6^ and then cocultured with different concentrations of DiD-labeled ABs in a 37 °C incubator for 4 h and 6 h. After removal at different time points and fixation with 4% paraformaldehyde, cytoskeletal staining and nuclear staining were performed, and the cells were placed under a laser confocal microscope (Olympus SpinSR10, Shinjuku, TKY, Japan) for photographic observation.

### Effect of PCL scaffolds loaded with BMSC-ABs on cell viability

The cytotoxicity of PCL-ABs and PCL to RAW264.7 cells was evaluated according to the instructions of the cell counting kit-8 (CCK-8; Beyotime Biotechnology). Cells (1 × 10^4^ cells/well) were first inoculated in 96-well plates and cultured overnight in DMEM. Next, BMSC-ABs (0, 5, 10, 15, 25, 50, 100, and 200 μg/mL) were added to the cells and incubated at 37 °C with 5% CO_2_ for 24 h. Then, the cells were washed with PBS and incubated with 10% CCK-8 solution at 37 °C for 4 h. Finally, the cells were incubated for 4 h using an enzyme marker (Bio-Rad, Hercules, CA, USA) to measure the absorbance at 450 nm.

### Western blotting analysis

Murine-derived RAW264.7 macrophages were treated with purified ABs, and after 0 h, 24 h, 48 h, and 72 h of culture, the cells were lysed, the proteins were extracted, the protein concentrations of the cells and ABs were determined by BCA protein assay, and the expression of the respective inflammatory factor proteins was detected by western blotting. Equal amounts of total proteins were separated by 4%–10% SDS‒PAGE and transferred to PVDF membranes, which were blocked with 5% skim milk at room temperature for 60 min and then incubated with primary antibodies (GAPDH, CD206, arginase-1, and VEGF) (1:1000) (rabbit mAb, Cell Signaling Technology) at 4 °C overnight. After washing with Tris-buffered saline with Tween® 20, the membranes were incubated with secondary horseradish peroxidase-coupled goat anti-rabbit IgG (Cell Signaling Technology) at room temperature for 2 h. The protein bands were visualized with enhanced chemiluminescence (Thermo Fisher Scientific).

### Flow cytometry

After treatment of the purified ABs with murine-derived RAW264.7 macrophages, the macrophages stained after 0 h, 24 h, 48 h, and 72 h of culture were analyzed by flow cytometry (Thermo Fisher Scientific). The cells were stained and analyzed using FITC-conjugated anti-mouse/human CD11b mAb (Blue Laser 488 nm), PE-conjugated anti-mouse CD86 mAb (Blue Laser 488 nm, Green Laser 532 nm/Yellow‒Green Laser 561 nm), Brilliant Violet 421-conjugated anti-mouse F4/80 mAb (Violet Laser 405 nm), and BrilliantViolet 650-conjugated anti-mouse CD206 mAb (Violet Laser 405 nm) (BioLegend, Diego, CA, USA) according to the manufacturer's instructions. All the data were analyzed using FlowJo software (Treestar Inc., Leonard Herzenberg, Palo Alto, CA, USA).

### RNA sequencing analysis

Total RNA was extracted from BMSC-derived ABs using TRIzol reagent (Invitrogen, Carlsbad, CA, USA). RNA extraction was followed by DNA digestion with DNaseI. RNA quality was determined using a NanodropTM OneC spectrophotometer (Thermo Fisher Scientific, Inc.) to determine the A260/A280 ratio. RNA integrity was confirmed by 1.5% agarose gel electrophoresis. The quality of the RNA was quantified with a Qubit 3.0 (Thermo Fisher Scientific, Inc.) using a QubitTM RNA wide range detection kit (Life Technologies). Strand RNA sequencing libraries were prepared using the Ribo-Off rRNA Depletion Kit (mouse) and the KC DigitalTM Strand mRNA Library Preparation Kit (Illumina, San Diego, CA, USA) according to the manufacturer's instructions. Library products corresponding to 200–500 bp were enriched, quantified, and finally sequenced on a NovaSeq 6000 sequencer (Illumina) using the PE150 model.

### Luciferase reporter assay

The sequences of CCL-1 (C-C motif chemokine ligand 1) containing the wild-type (WT) or mutant (Mut) binding site of miR-21a-5p were designed and synthesized by GenePharma (Shanghai, China). 293 T and RAW264.7 cells were cotransfected with the corresponding plasmids and miR-21a-5p mimics/miR-NC or miR-21a-5p inhibitors/inh-NC with Lipofectamine 2000 (Invitrogen, Carlsbad, CA, USA). To construct a luciferase reporter gene vector containing the CCL-1 promoter, full-length CCL-1 promoters containing wild-type or mutant CCL-1 were cloned and inserted into pGL3-basic vectors (Genecreate, Wuhan, China) and subsequently cotransfected with or without the miRNA overexpression vector. After 48 h of incubation, the activities of firefly and renilla luciferase were measured using the dual luciferase reporter assay kit (Promega, Madison, WI, USA).

### Analysis of the effect of PCL-loaded bone marrow MSC-derived apoptotic vesicles on M0 macrophage programming after microRNA transfection

The miR-21a-5p inhibitor and inhibitor-negative control (NC) (Shanghai Gene Pharma Co., Ltd., Shanghai, China) were used at a final concentration of 100 nM Lipofectamine 3000 (Invitrogen, Thermo Fisher Scientific, Inc.). The sequences of the inhibitor and negative control are shown in Table 1. The same conditions were applied for each transfection experiment. After 12 h, the transfection was assessed under a fluorescence microscope, and further experiments were continued at 24 h.

**Table 1:** Primer sequences for miR-21a-5p and the inhibitor.

DUplexName	SenseSeq5'→3′	SenseSeq5'→3′	MW
mmu-miR-21a-5p	UAGCUUAUCAGACUGAUGUUGA	(FAM)AACAUCAGUCUGAUAAGCUAUU	14512.99
mmu-miR-21a-5p inhibitors	(Cy3) (mU) (mC) (mA) (mA) (mC) (mA) (mU) (mC) (mA) (mG) (mU) (mC) (mU) (mG) (mA) (mU) (mA) (mA) (mG) (mC) (mU) (mA)		7909.72
Inhibitors-NC	(mC) (mA) (mG) (mU) (mA) (mC) (mU) (mU) (mU) (mU) (mG) (mU) (mG) (mU) (mA) (mG) (mU) (mA) (mC) (mA) (mA)		6953.66

After the transfection of the miR-21a-5p inhibitor and CCL-1 receptor antagonist into RAW264.7 macrophages, the expression of CD206, arginase-1, and CCL-1 mRNA was assessed by quantitative PCR at 24 h. Total RNA was isolated using TRIzol reagent (Life Technologies). According to the manufacturer's instructions (Promega, Madison, WI, USA), single-stranded cDNA was prepared from 1 μg of mRNA using reverse transcriptase with oligomeric dT primers and V-normalized to GAPDH mRNA levels, and the respective inflammatory factor gene expression was determined using the 2^−ΔΔCt^ method. The primer sequences for the RAW264.7 macrophages are shown in Table 2. The effect of bone marrow MSC-derived apoptotic vesicles on the reprogramming of M0 macrophages was assessed by western blot analysis using anti-CD206, anti-arginase-1, and anti-CCL-1 primary antibodies and GAPDH as an internal reference at 48 h.

**Table 2:** Primer sequences for quantitative reverse transcription PCR.

Primers	Sequence (5'−3′)
CD206-F	CTCTGTTCAGCTATTGGACGC
CD206-R	TGGCACTCCCAAACATAATTTGA
Arginase-1-F	CGGCAGTGGCTTTAACCTTG
Arginase-1-R	TTCATGTGGCGCATTCACAG
CCL-1-F	GATGAGCCACCTTCCCATCC
CCL-1-R	TGACTGAGGTCTGTGAGCCT
GAPDH-F	ACTCTTCCACCTTCGATGCC
GAPDH-R	TGGGATAGGGCCTCTCTTGC

### Cytokine measurements in programmed M2 macrophages

The cells were cultured in serum-free DMEM for 24 h, after which 500 μL of medium was collected. The samples were processed using the Bio-Plex Mouse Cytokine 23-Plex Panel Array (Bio-Rad Laboratories, Hercules, CA, USA) and assayed using the Bio-Plex Protein Array System (Bio-Rad Laboratory) according to the manufacturer's instructions. Concentrations were calculated using the following equation: relative concentration = cytokine concentration ÷ total protein concentration. Additionally, the levels of the cytokines TNF-α, TGF-β, von Willebrand factor (vWF), and VEGF were measured using a mouse ELISA kit (BioVision, San Francisco Bay, CA, USA), standard curves were generated according to the manufacturer's instructions, and the concentrations of the factors were determined from the optical density data.

### Effect of programmed M2 macrophages on fibroblasts

NIH-3T3 mouse fibroblasts were inoculated in the lower layer of a Transwell plate (6-well plate, 0.4 μm, Jet Bio-Filtration, Guangzhou, China) and cultured to 100% confluence. RAW264.7 cells treated in the PBS group (M0), ABs group (M0 + ABs), PCL group (M0 + PCL), and PCL + ABs (M0 + PCL + ABs) groups were transferred to the upper layer of the Transwell plate at 30%–40% inoculation density, and when the cell density reached 100%, the old medium was removed, the cells were washed with PBS three times, and the medium was replaced with fresh complete culture medium. NIH-3T3 cells were scratched vertically along the diameter of the 6-well plate using a 200 μL pipette tip, and RAW264.7 cells treated as described above were placed on the upper layer of the Transwell plate for coculturing. After 12 h, the scratch area of each group was recorded, and the change in scratch area was calculated by ImageJ software (NIH, Bethesda, MD, USA).

### Establishment of a C57BL/6 mouse model of trauma

Twelve male (8-week-old) C57BL/6 mice (18–22 g) were purchased from the Animal Experiment Center of Chongqing Medical University (Chongqing, China). The mice were randomly divided into three groups, the PBS group, PCL group, and BMSC-AB-PCL group, with four mice in each group. After anesthesia by intraperitoneal injection of sodium pentobarbital (40 mg/kg), the mice were shaved and a full-thickness skin wound (0.8 cm in diameter) was produced on the back of each mouse. PCLs and PCLs loaded with BMSC-ABs were then placed over the wound surface of the mice. Images of the wounds were taken on days 0, 2, 4, 6, 8, and 10. Changes in wound size were analyzed using Image-Pro Plus software (Media Cybernetics, Rockville, MD, USA).

### Distribution of PCL fibrous scaffolds of BMSC-ABs on trabeculae

ABs were fluorescently labeled according to the manufacturer's reagent instructions (Cyanine7 NHS Ester Cy7 NHS, MKBio, Shanghai, China). *In vivo* fluorescence analysis was performed on the trabeculae of C57BL/6 mice. ABs (10 μg/10 μL) or PBS was injected into the subcutaneous tissue of mice (*n* = 3), and the fluorescence intensity was measured by an *in vivo* imaging system (AniView100, BLT, Guangzhou, China). The fluorescence intensity of the region (ROI) was quantified using AniView software (BLT).

### Histological analysis

Mice were sacrificed by intraperitoneal injection of 150 mg/kg sodium pentobarbital on day 10 after the establishment of the mouse model of trauma. Mouse tissues were collected to observe pathological changes. Tissues from the wound site were collected, fixed in 10% paraformaldehyde, and embedded in paraffin. Pathological changes in the tissues were examined using an hematoxylin-eosin staining kit (Solarbio, Beijing, China). Semiquantitative analysis of hematoxylin-eosin staining was based on the number of follicles and granulation tissue (scores: 0–4; higher scores indicate greater numbers), inflammatory infiltration (scores: 0–2; higher scores indicate less infiltration), and neovascularization (scores: 0–4; higher scores indicate more vascularity). An overall score was calculated, with higher scores indicating better wound recovery. In addition, collagen deposition in the tissue was measured using a Masson trichrome staining kit (Solarbio) and quantified using ImageJ software. Neovascularization was further examined by immunohistochemical staining with a CD31 (rabbit mAb, 1:2000) (Abcam, Cambridge, England, UK) antibody. All the quantitative analyses were performed independently by three pathologists in random unknown groups.

### Statistical analysis

All the data are shown as mean ± standard deviation. All groups were compared using *t*-tests or one-way ANOVA. *p* values less than 0.05 were considered statistically significant. Graphical analysis was performed using GraphPad Prism 9.0 (GraphPad Software, San Diego, CA, USA).

## Results

### Acquisition and identification of bone marrow MSC-derived ABs and preparation and characterization of PCL fibrous scaffolds

**Figure 1:**
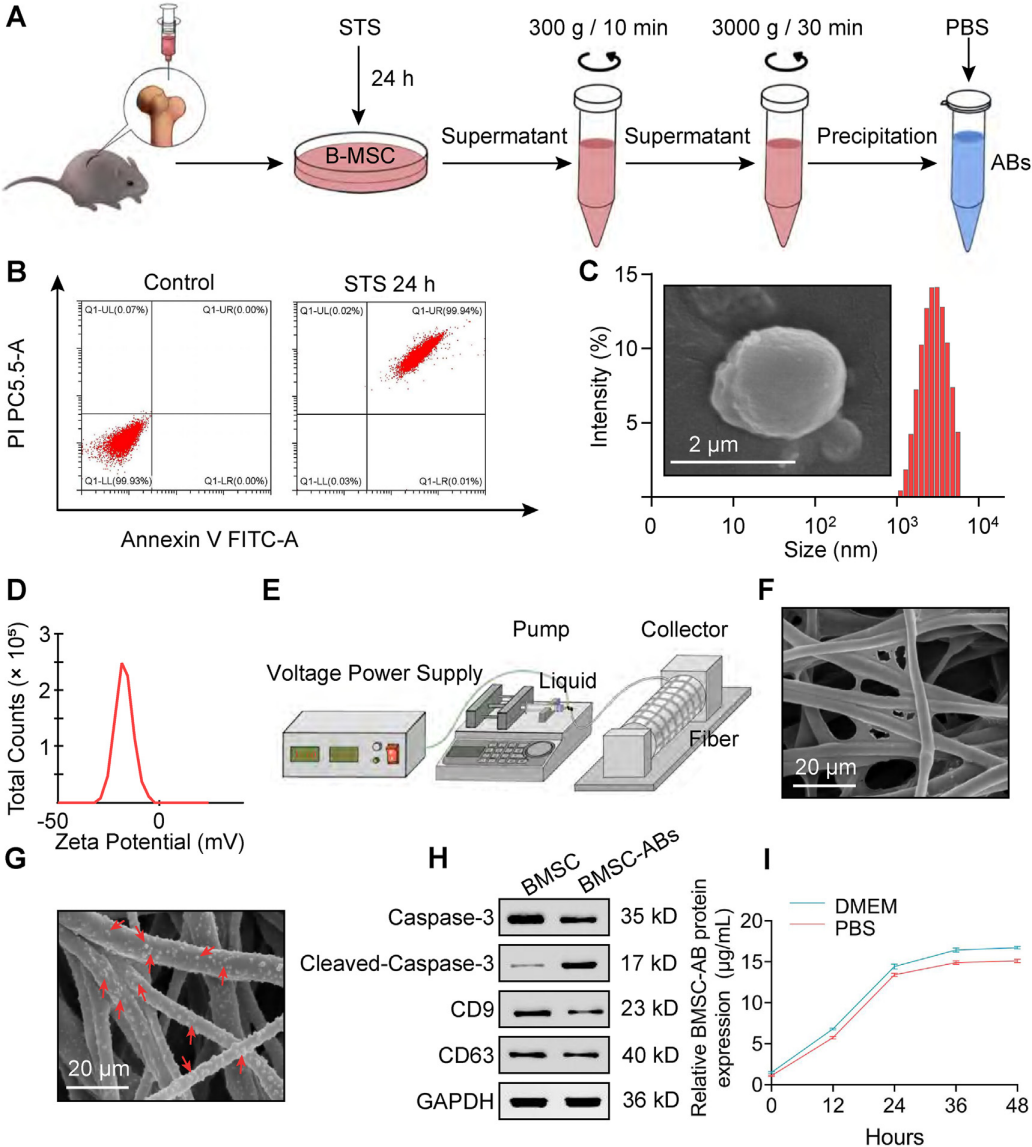
Acquisition and identification of bone mesenchymal stem cell-derived apoptotic bodies (BMSC-ABs) and preparation and characterization of polycaprolactone (PCL) fiber scaffolds. **(A)** Acquisition of BMSC-ABs. **(B)** Identification of apoptotic cells by flow cytometry. **(C)** Morphological images of ABs. Left: scanning electron microscopy (SEM) images of ABs (scale bar, 2 μm); right: particle size of ABs measured by dynamic light scattering (DLS). **(D)** Zeta potential of the ABs. **(E)** Flow chart of PCL scaffolds prepared by electrostatic filament mimicking technique. **(F)** SEM image of an electrostatic filament mimicking the PCL scaffold. Scale bar, 20 μm. **(G)** SEM images of electrostatic filament-mimicking PCL scaffolds loaded with ABs. Red arrows indicate sites representing ABs. **(H)** Identification of ABs by western blot. **(I)** Release rate of AB-loaded PCL scaffolds.

We obtained primary bone marrow MSCs from the femurs and tibias of 6-to-8-week-old male C57BL/6 mice, and after induction of the cells with staurosporine in culture for 24 h, apoptosis was detected by flow cytometry, which revealed a 99.94% apoptosis rate in the control group (Fig. 1B). MSC-derived ABs were collected by differential gradient centrifugation (Fig. 1A). The collected ABs showed a typical vesicle structure under scanning electron microscopy, and dynamic light scattering analysis revealed a particle size of approximately 2.314 μm (Fig. 1C). The zeta potential of the ABs was −17.8 mV, indicating that they were relatively stable (Fig. 1D). In addition, western blotting confirmed that the ABs in the collected pellets all expressed the same membrane markers (CD9 and CD63) as the source cells and had high levels of cleaved cysteine protease-3 (Fig. 1H). These results indicate the successful collection of apoptotic vesicles while inducing apoptosis.

Next, we prepared PCL scaffolds by electrostatic filament mimicry (Fig. 1E) and observed them by scanning electron microscopy (Fig. 1F). The extracted MSC-ABs were loaded on the PCL scaffold, and subsequent scanning electron microscopy showed that the ABs were successfully adsorbed on the scaffold surface (Fig. 1G). To determine the release rate of the AB-loaded PCL scaffolds, the ABs were placed in PBS and DMEM, and the suspensions were collected every 12 h. The protein concentration was detected by BCA, and the results showed that the release rate tended to stabilize after 24 h, and the release amount in DMEM was slightly greater than that in PBS (Fig. 1I). These results indicate that the electrostatic filament-mimetic preparation of PCL scaffolds can better deliver and release apoptotic vesicles.

**Figure 2:**
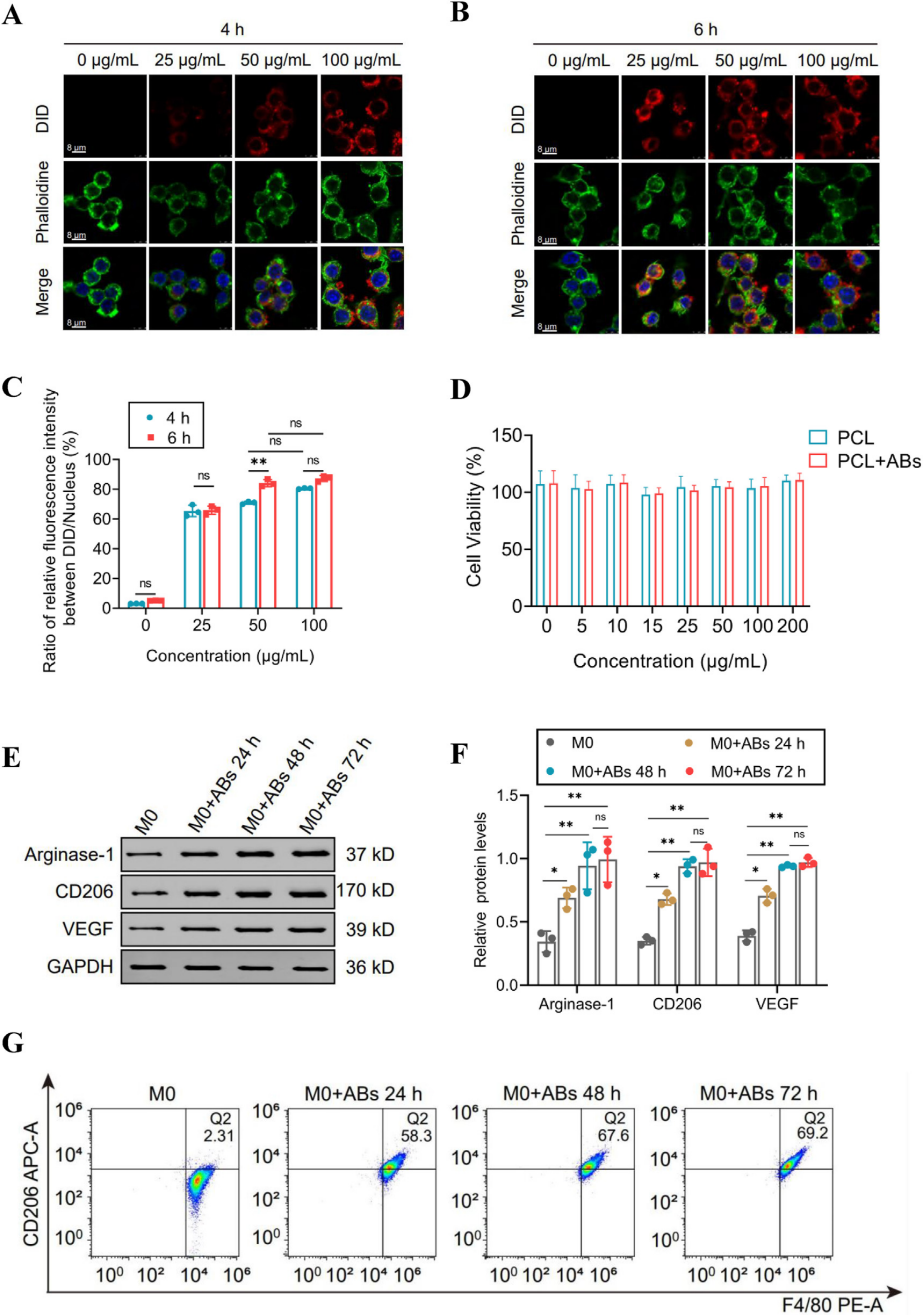
Polycaprolactone-loaded bone mesenchymal stem cell-derived apoptotic bodies (PCL-BMSC-ABs) guided *in vitro* polarization of M0 to M2 macrophages (Mϕs). **(A)** Immunostaining of 0–100 μg/mL PCL-BMSC-ABs incubated for 4 h. Green: cytoplasm; blue: nucleus; red: ABs. **(B)** Immunostaining of 0–100 μg/mL PCL-ABs incubated for 6 h. Green: cytoplasm; blue: nucleus; red: ABs. **(C)** Relative fluorescence intensity of 0–100 μg/mL PCL-BMSC-ABs incubated for 4 h and 6 h *n* = 3; ^∗∗^*P* < 0.01. **(D)** RAW264.7 cell viability after incubation with PCL-ABs. **(E)** Western blot analysis of Mϕs reprogrammed with the M2 phenotype treated with 50 μg/mL PCL-ABs. **(F)** Relative grayscale values of western blot analysis of 50 μg/mL PCL-BMSC-ABs Mϕs reprogrammed with the M2 phenotype. *n* = 3; ^∗∗^*P* < 0.01, ^∗^*P* < 0.05. **(G)** Flow cytometry comparison of CD206-positive M1 Mϕs and Mϕs incubated with 50 μg/mL PCL-BMSC-ABs for different periods.

Moreover, we evaluated the efficiency of macrophage uptake of apoptotic vesicles from bone marrow-derived MSCs by incubating macrophages with PCL scaffolds of fluorescent dye-labeled apoptotic vesicles at concentrations ranging from 0 to 100 μg/mL for 4 or 6 h (Fig. 2A, B). Confocal microscopy revealed that the attachment and internalization of apoptotic vesicles from MSCs increased in a dose- and time-dependent manner, with the internalization of apoptotic vesicles by macrophages stabilizing at an apoptotic vesicle concentration of 50 μg/mL for 6 h (Fig. 2C). The survival rate of RAW264.7 cells incubated with PCL and PCL + ABs was maintained at approximately 90%, as determined by CCK-8 assays (Fig. 2D). Therefore, 50 μg/mL was chosen as the optimal concentration of apoptotic vesicles to determine whether reprogramming of the macrophage phenotype was possible, and the rate was maximized at 6 h.

### Evaluation of the polarization of M0 to M2 macrophages by PCL-loaded BMSC-ABs *in vitro*

To test whether BMSC-ABs could induce M0 macrophages to polarize to an anti-inflammatory M2 phenotype, we first incubated RAW264.7 cells with 50 μg/mL PCL-BMSC-ABs for 0 h, 24 h, 48 h, and 72 h. Western blot analysis revealed increased expression of arginase-1 and CD206, indicating that BMSC-ABs enhanced the transformation of M0 macrophages to an anti-inflammatory M2 phenotype and that VEGF expression also increased, indicating that BMSC-ABs promoted angiogenesis (Fig. 2E). Notably, the expression of arginase-1, VEGF, and CD206 was significantly increased in RAW264.7 cells incubated with BMSC-ABs and remained stable at 48 h (Fig. 2F). To accurately quantify the extent of M0 polarization to M2 polarization, we used flow cytometry to compare the CD206 positivity of RAW264.7 cells incubated with 50 μg/mL BMSC-ABs for different durations (Fig. 2G). Flow cytometry analysis revealed that the percentage of M0 macrophages that reprogrammed into M2 macrophages reached 58.3% after incubation with BMSC-ABs for 24 h, increased significantly to 67.6% after 48 h, and stabilized at 69.2% after 72 h (Fig. S1B).

### PCL-loaded BMSC-ABs drive the polarization of M0 to M2 macrophages through miR-21a-5p

**Figure 3:**
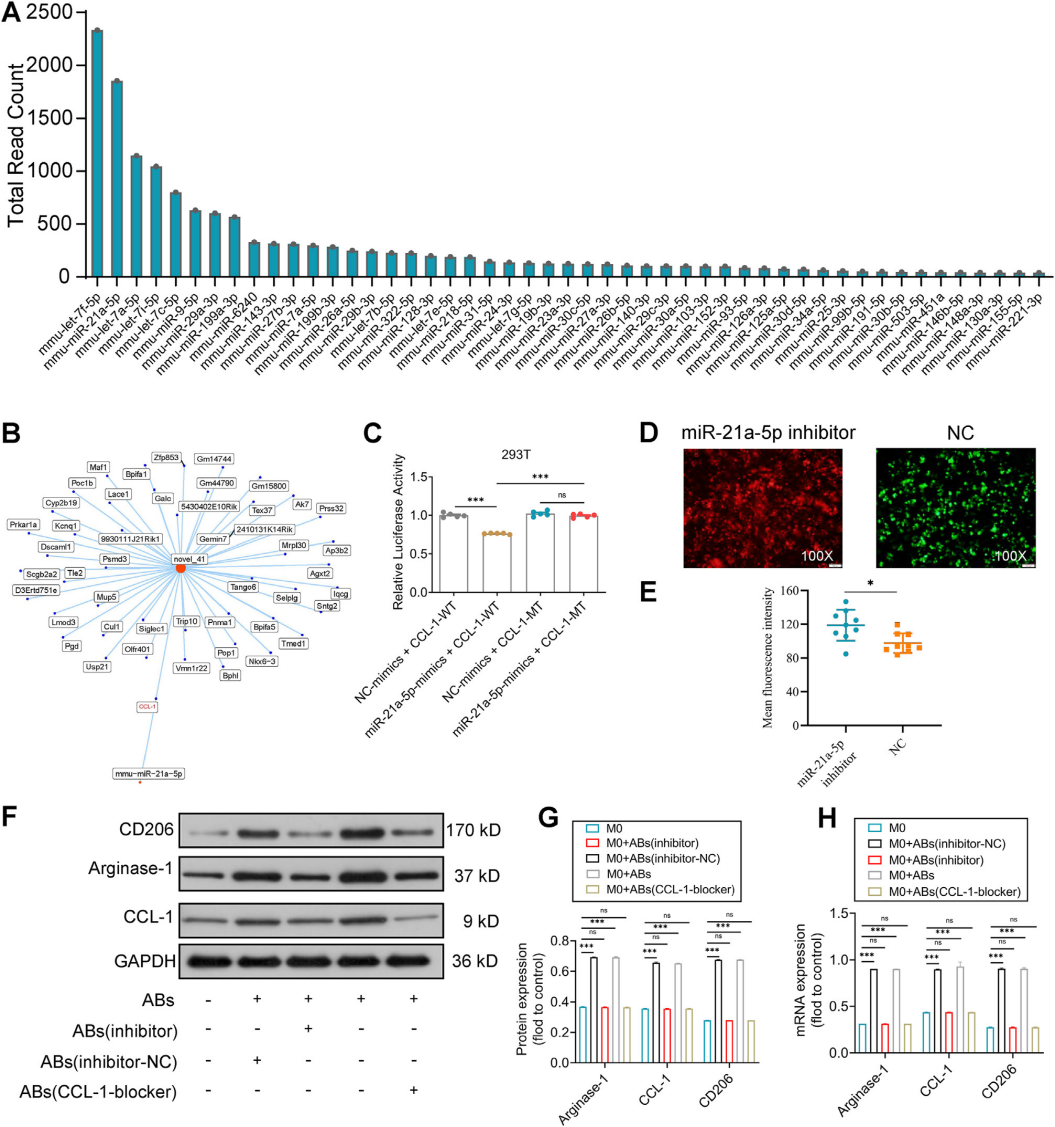
miRNA sequencing analysis of bone mesenchymal stem cell-derived apoptotic bodies (BMSC-ABs)-loaded polycaprolactone (PCL) scaffolds driving the molecular reprogramming of macrophages (Mϕs) to M2-Mϕs. **(A)** The top 50 known miRNAs detected in BMSC-ABs. **(B)** miRNA-mRNA regulatory network. **(C)** The binding of miR-21a-5p to the target gene CCL-1 in 293T cells validated by dual luciferase assay. **(D)** Colocalization of miR-21a-5p with Mϕs. **(E)** Fluorescence intensity analysis. *n* = 3; ^∗^*P* < 0.05. **(F)** Western blot analysis of the effect of the miR-21a-5p inhibitor on the reprogramming of BMSC-ABs to Mϕs. **(G)** Relative grayscale values of the western blot analysis of miR-21a-5p inhibitor's effect on the reprogramming of BMSC-ABs that drive Mϕs to M2-Mϕs. *n* = 3; ^∗∗∗^*P* < 0.001. **(H)** Quantitative PCR analysis of miR-21a-5p inhibitor's effect on the reprogramming of BMSC-AB-driven Mϕs to M2-Mϕs. *n* = 3; ^∗∗∗^*P* < 0.001.

Extracellular vesicles can regulate gene expression at the posttranscriptional level by delivering miRNAs, thereby affecting the function of recipient cells. We analyzed and quantified the expression of miRNAs in BMSC-ABs. A total of 353 known miRNAs were identified via miRNA sequencing analysis of RNA purified from BMSC-ABs. Next, the top 50 known miRNAs detected in BMSC-ABs were sorted based on total read counts (Fig. 3A). After the miRNAs were predicted to target genes, miR-21a-5p was predicted to bind to the target gene CCL-1 by dual-luciferase experiments (Fig. 3B, C; Fig. S1A). After transfection, colocalization of the fluorescently labeled miR-21a-5p inhibitor and macrophages was detected by fluorescence microscopy (Fig. 3D, E). BMSC-ABs were added to M0 that had been transfected with miR-21a-5p inhibitors, and CCL-1 receptor blockers were added to M0 that had been transfected with miR-21a-5p inhibitors, and the transcript levels of CD206, arginase-1, and CCL-1 were assessed at 48 h. The effect of stem cell-derived ABs on the programming of M0 was assessed by western blot analysis after 48 h. The western blot results revealed a significant increase in M2-specific proteins detected in the BMSC-AB and miR-21a-5p inhibitor NC groups compared with the control, miR-21a-5p inhibitor, and CCL-1 receptor blocker groups (Fig. 3F), indicating that miR-21a-5p in BMSC-ABs significantly affected macrophage function via CCL-1. The western blot results were consistent with the quantitative PCR results (Fig. 3F–H).

### Analysis of the *in vitro* anti-inflammatory and fibroblast migration-promoting effects of programmed M2 macrophages *in vitro*

**Figure 4:**
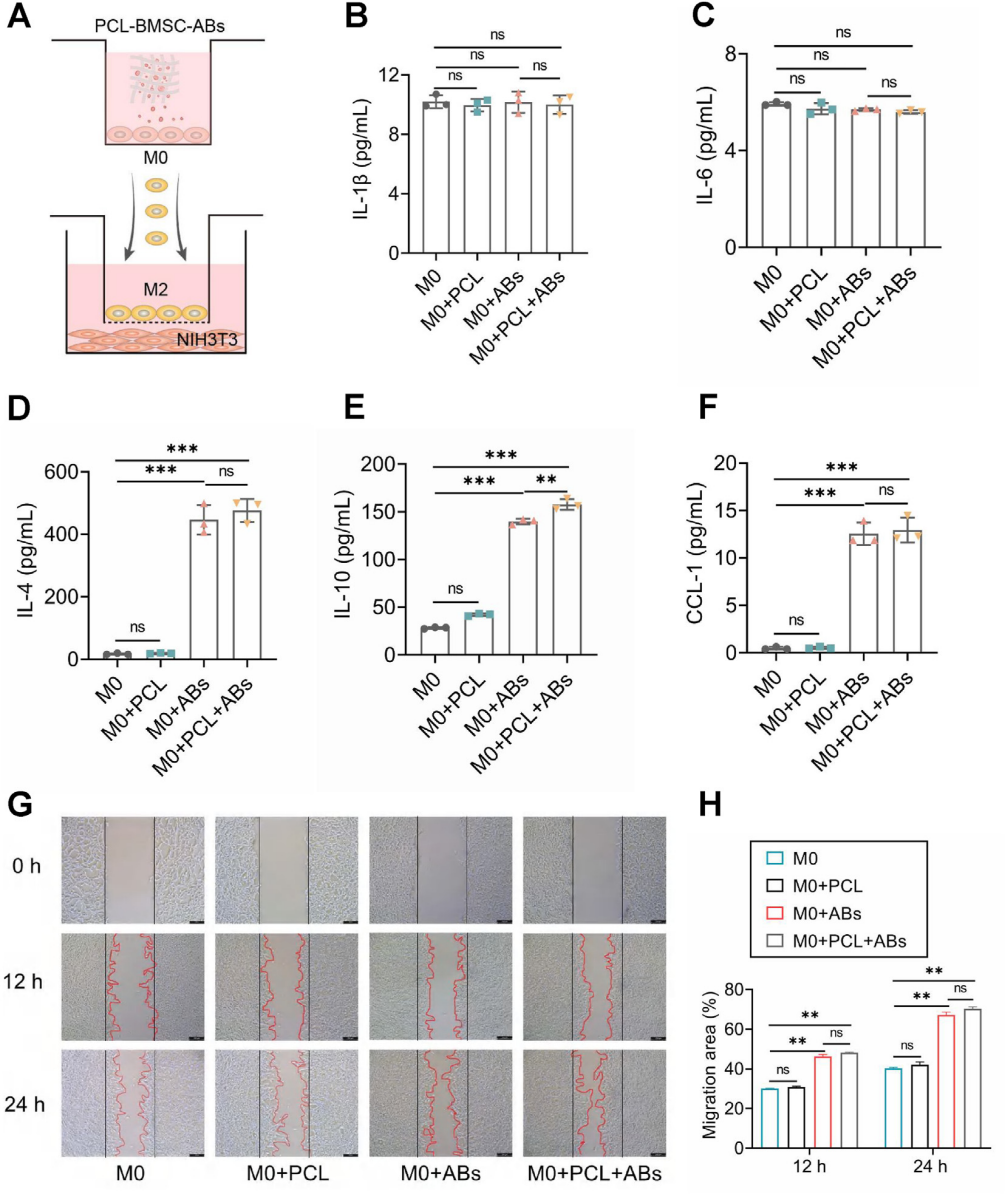
*In vitro* anti-inflammatory and pro-fibroblast migration effects of reprogrammed polycaprolactone (PCL)-loaded M2 macrophages (RM2). **(A)** Schematic diagram of the scratch assay. **(B–F)** Levels of cytokines and chemokines in the supernatants of various cell types. *n* = 3; ^∗∗∗^*P* < 0.001, ^∗∗^*P* < 0.01. **(G)** Differences in fibroblast migration at different time points (with 4 × microscopy). **(H)** Migration distance of fibroblasts at different time points. *n* = 3; ^∗∗^*P* < 0.01.

Although the above findings suggest that BMSC-ABs can drive the transition of M0 to M2 phenotype via miRNA (Figure 2, Figure 3), the ability of programmed M2 macrophages to produce anti-inflammatory cytokines and promote fibroblasts is unknown. We evaluated changes in the secretion of anti-inflammatory and pro-inflammatory cytokines by programmed M2 macrophages and activated M0 macrophages in serum-free medium and then further analyzed the effect of programmed M2 macrophages on fibroblast migration (Fig. 4; Fig. S1). A Bio-Plex protein array was used to analyze the levels of certain cytokines and chemokines in the supernatants of various cell types. In the M0 and M0+PCL groups, the levels of anti-inflammatory cytokines (IL-4, IL-10, CCL-1, and TGF-β) and vascular indicators (VEGF and vWF) were significantly lower than those in the M0+Abs and M0+PCL + ABs groups, and the expression levels of pro-inflammatory factors in each group (IL-1β, IL-6, and TNF-α) were not significantly different (Fig. 4B–F; Fig. S1C–F). Interestingly, there was a significant difference in the anti-inflammatory cytokine IL-10 and the angiogenic indicator vWF between the M0+ABs and M0+PCL + ABs groups, suggesting that the PCL material may have a synergistic role in the transition of M0 to M2 phenotype. Different macrophage populations were cocultured with fibroblasts in transwell chambers for 24 h (Fig. 4A), and differences in fibroblast migration were observed by inverted microscopy at 4× every 12 h (Fig. 4G). The results showed that fibroblasts reached satisfactory migration capacity within 24 h. The M0+PCL + Abs and M0+ABs groups demonstrated significantly enhanced fibroblast migration compared with the M0 and M0+PCL groups (Fig. 4H). These results further support the idea that BMSC-ABs can program M0 to M2 and may promote wound healing.

### A mouse trauma model validates that PCL-loaded BMSC-ABs exert bidirectional anti-inflammatory and angiogenic effects via macrophage programming to promote trauma healing

**Figure 5:**
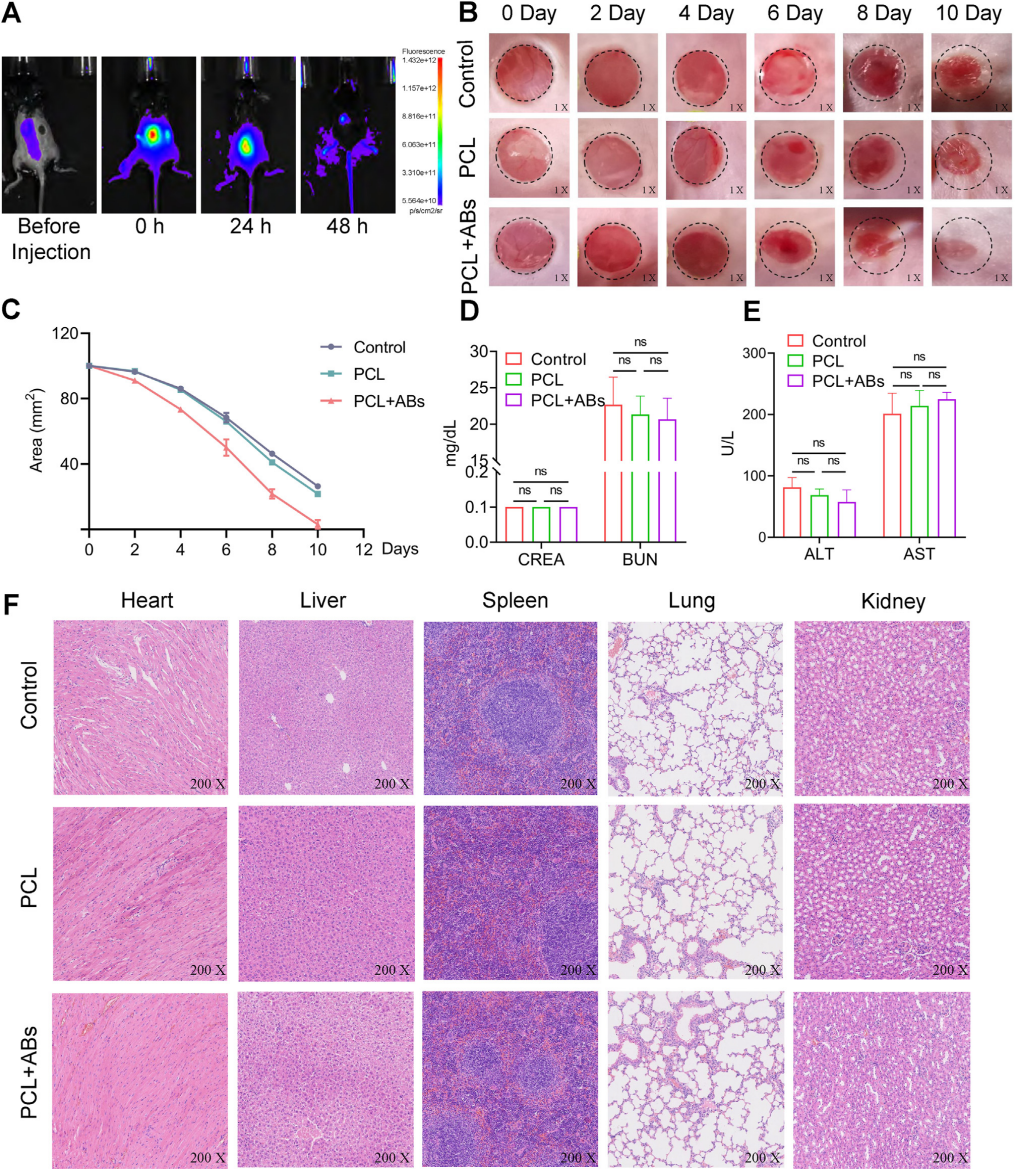
*In vivo* biodistribution of bone mesenchymal stem cell-derived apoptotic bodies (BMSC-ABs). **(A)** Real-time imaging of Cy7-N-hydroxysuccinimide (NHS)-labeled ABs. Fifty micrograms of apoptotic vesicles suspended in 20 μL of phosphate buffer saline solution were injected into the tissue near the wound site through subcutaneous injection for real-time observation. **(B)** Observation of trauma-related changes in mice at different time points. **(C)** Wound size changes in mice at different time points. **(D)** Kidney function indicators. **(E)** Liver function indicators. **(F)** Histopathological sections of various tissues and organs.

Before exploring the effect of PCL-loaded BMSC-ABs on wound progression, we performed real-time fluorescence imaging analysis of the *in vivo* distribution of PCL-loaded BMSC-ABs. The fluorescence signal of Cy7-N-hydroxysuccinimide (NHS)-labeled ABs was clearly maintained after trauma for 2 days and gradually decreased over time. On day 2 after coinjection, the signal decreased to less than 10% of the initial value. Substantial programming was found at 48 h during *in vitro* coincubation (Fig. 5A; Fig. S1G). This result suggested that locally injected ABs will have sufficient time to reprogram M0 to M2. Thus, our data suggest that local macrophage programming can be achieved every two days of local treatment.

To investigate the effect of BMSC-ABs on trauma-related inflammation and angiogenesis, we generated a 0.8 cm diameter trauma model in the skin of anesthetized mice on the back via a trauma punch and covered the trauma with PCLs loaded with BMSC-ABs and a separate PCL. Wound healing assays revealed that the PCL-BMSC-ABs promoted wound healing (Fig. 5B), and there was no significant difference in wound healing between the PCL and PBS groups (Fig. 5C). Moreover, the liver and kidney function indices and histopathological sections of the mice were not significantly different between the groups (*P* > 0.05) (Fig. 5D–F).

**Figure 6:**
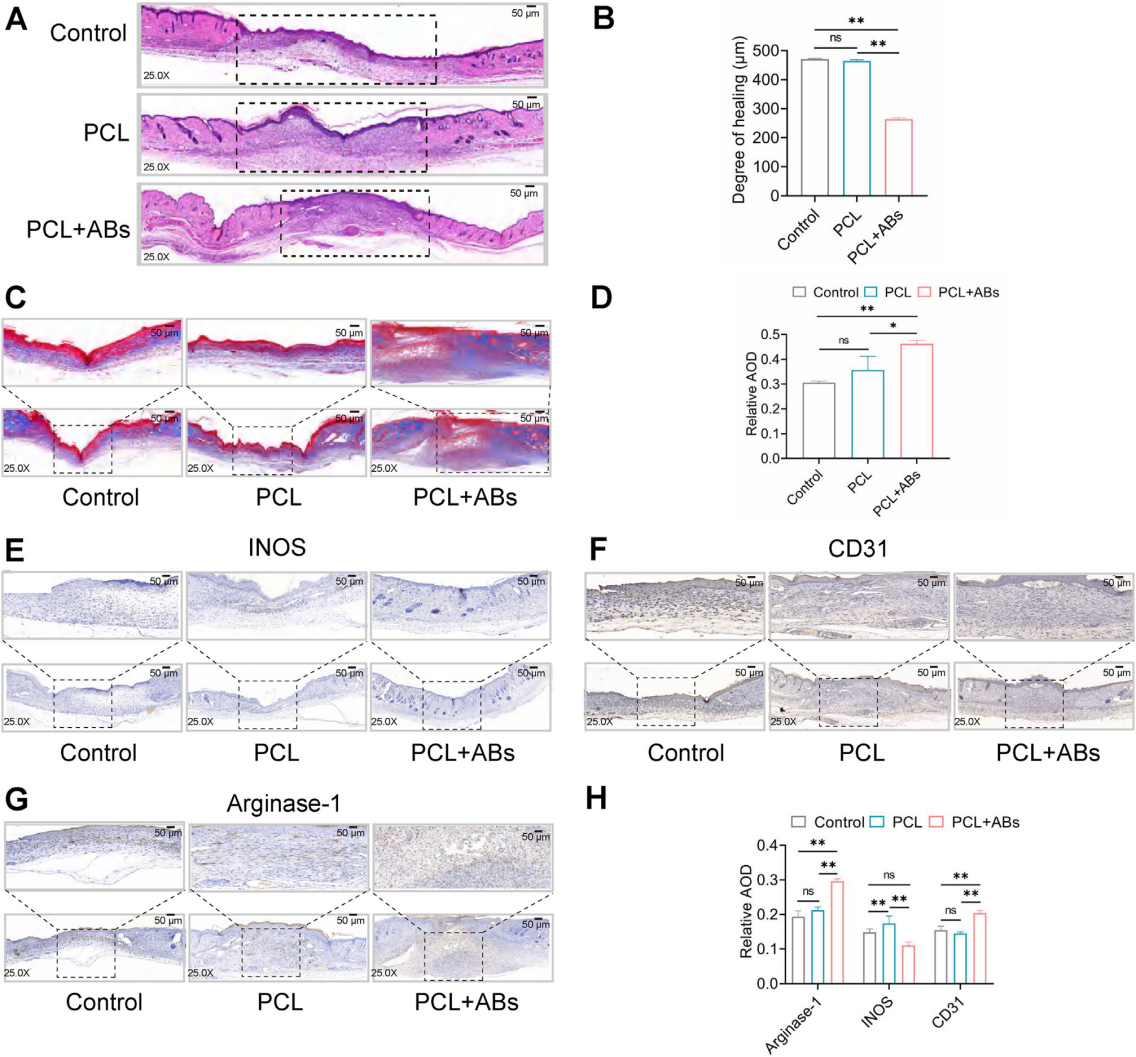
Effect of bone mesenchymal stem cell-derived apoptotic bodies (BMSC-AB)-loaded polycaprolactone (PCL) scaffolds on wound healing. **(A)** Hematoxylin-eosin staining maps of each group. **(B)** Degree of healing shown by hematoxylin-eosin staining. ^∗∗^*P* < 0.01. **(C)** Masson staining of each group. **(D)** Masson staining of each group relative to the average optical density (AOD). Differences between the PCL group and the PCL + AB group. ^∗∗^*P* < 0.01, ^∗^*P* < 0.05. **(E)** Immunohistochemical analysis of the expression of the trabecular cell marker inducible nitric oxide synthase (INOS). **(F)** Immunohistochemical analysis of the expression of the traumatic cell marker CD31. **(G)** Immunohistochemical analysis of the traumatic cell marker arginase-1. **(H)** Quantitative analysis of traumatic cell markers. Differences between the PCL + ABs group and each other group. ^∗∗^*P* < 0.01.

We also examined the histological changes in the wounds. The results of hematoxylin-eosin staining (Fig. 6A, B) and Masson staining (Fig. 6C) showed that the wounds in the PCL-ABs treatment group exhibited a significant trend toward healing, inflammatory cell regression, and collagen fiber formation (*P* < 0.05), while those in the control group (PBS injection) and the PCL treatment group covered with PCL alone showed a significant trend toward delayed wound healing compared with those in the PCL-ABs group and increased inflammatory cells, and there was no significant difference between the two groups (*P* > 0.05) (Fig. 6D). Based on these histological findings, we further evaluated the distribution of traumatic macrophage types by immunohistochemistry staining of the traumatic tissue. The percentage of arginase-positive macrophages significantly increased (*p* < 0.01) in the PCL-ABs treatment group (Fig. 6G, H), whereas the percentage of INOS (inducible nitric oxide synthase)-positive macrophages decreased (*P* < 0.01) (Fig. 6E–H), indicating that ABs can promote the conversion of macrophages from the M0 phenotype to the M2 phenotype. Interestingly, the percentage of CD31-positive cells increased in the PCL-AB treatment group (*P* < 0.05) (Fig. 6F–H), suggesting that ABs may promote neovascularization. The above results further demonstrate that BMSC-derived apoptotic vesicles can reduce inflammatory infiltration by programming M0 macrophages into M2 macrophages, thereby preventing or reducing delayed wound healing and thus exerting anti-inflammatory and angiogenic effects in mice.

## Discussion

The skin is the body's first line of defense and has the essential function of repelling pathogens and preventing mechanical, chemical and physical damage, which, when damaged, can also lead to infection and necrosis, as well as other serious local and systemic consequences.^25^ Persistent skin inflammation can lead to the onset and progression of chronic inflammatory diseases, resulting in delayed wound healing.^26^ Therefore, there is an urgent need for novel effective strategies for the treatment of skin injuries to improve the healing process and repair the skin barrier.^27^ An imbalance in macrophage number and function is one of the important causes of the long-term persistence of trauma in the inflammatory phase.^28^ A decrease in the number of M2 macrophages leads to a significant increase in the levels of the traumatic local inflammatory cytokines TNF-α and IL-6 and a decrease in the level of the anti-inflammatory cytokine IL-10.^29^^,^^30^ Given the high plasticity among macrophage phenotypes, restoring the normalization of macrophage phenotype number and function by directly reprogramming M0 macrophages to M2 macrophages may be an effective strategy for treating traumatic inflammation and accelerating wound healing.

The field of exosome research has attracted renewed interest due to the discovery of tubular RNAs, including mRNAs and miRNAs, in exosomes.^31^ Previous studies have shown that exosomes secreted by human MSCs accelerate wound healing by reducing the number of neutrophils, inhibiting macrophage recruitment to the site of injury, promoting M2 macrophage polarization, angiogenesis, and collagen deposition, and modulating the inflammatory response.^32^ However, the limitations of obtaining the number and function of exosomes under different experimental conditions, the highly variable relative ratio of cell-released exosomes to other small extracellular vesicles, the difficulty in controlling exosome production and release, and the nonspecific recognition of target cells still hamper clinical wound repair applications.^33^, ^34^, ^35^ In recent years, apoptotic vesicles have been found to be effective in overcoming the limitations of exosome application as a product of programmed apoptosis.^36^ Phosphatidylserine (PtdSer, PS) and annexin-V (Anxin-V) can be transferred to the surface of the vesicle envelope during apoptosis, where they act as signals to trigger phagocyte recognition and uptake and accurate delivery of apoptotic vesicles to their target cells for apoptosis-mediated cell reprogramming.^37^ During the acute inflammatory phase of wound healing, most of the removal of decayed and damaged cells is carried out by macrophages and neutrophils through phagocytosis and in the absence of an inflammatory response during this process. Apoptotic vesicles promote the efficient removal of apoptotic material by peripheral phagocytes and mediate the transfer of biomolecules, including miRNAs and proteins, between cells to aid intercellular communication.^38^, ^39^, ^40^ There is evidence that mononuclear phagocytes respond to apoptotic cells by releasing anti-inflammatory factors, including IL-10 and TGF-β1 and that apoptotic vesicles can break down apoptotic cells into smaller fragments to facilitate the removal of apoptotic debris and intercellular communication.^41^, ^42^, ^43^ Extensive apoptosis of exogenous MSCs in a short period, down-regulation of the expression of the proinflammatory cytokines IL-6 and TNF-α, and up-regulation of the anti-inflammatory cytokine IL-10 in the wound area accelerate wound healing.^44^ Liu et al reported that ABs derived from bone marrow MSCs triggered the polarization of macrophages toward the M2 phenotype, which could enhance the migration and proliferation of fibroblasts.^18^ However, the molecular mechanisms by which stem cell-derived ABs act have not been elucidated. In this context, we extracted MSC-derived ABs and programmed them to verify the effect of ABs on wound healing trends, inflammatory cell regression, and collagen fiber formation in a mouse skin wound healing model.

In this study, PCL fiber scaffolds prepared by electrostatic spinning were used to deliver apoptotic vesicles to the trauma site. PCL, a biodegradable, biocompatible and FDA-approved polymer, is widely used in the fields of tissue regeneration and drug delivery because it better mimics the physical structure of the extracellular matrix and has suitable mechanical properties.^45^^,^^46^ Electrostatic spinning can provide nanoscale extracellular matrix mimetic structures with a high specific surface area and high porosity, which overcomes the disadvantages of conventional nanofibrous scaffolds due to their smaller fiber size and pore size, dense fiber structure, and lower porosity, resulting in low cell infiltration. More importantly, the porosity of PCL allows nutrient exchange between the inner and outer sides of the trauma surface, which is more compatible with the needs of trauma healing.^47^ Therefore, we loaded MSC-ABs onto PCL fiber scaffolds in this study. PCL scaffolds have good biocompatibility, and BMSC-derived apoptotic vesicle-loaded PCL scaffolds could prevent or reduce delayed wound healing by reprogramming M0 macrophages into M2 macrophages and reducing inflammatory infiltration, which in turn exerted anti-inflammatory and angiogenic effects in mice. However, the exact underlying mechanism needs to be further investigated.

miRNAs are a class of small noncoding RNAs approximately 22 nt long that are widely found in plants and animals and mainly function by inhibiting and regulating the translation of target genes.^48^ The genome of an organism can encode thousands of miRNAs, which target approximately 60% of protein-coding genes, and regulate gene expression translation by binding to target mRNAs, leading to inactivation or activation of the latter and participating in important biological processes in life, such as cell proliferation, differentiation, apoptosis, growth and development of the organism, and regulation of the immune response to pathogen infection.^49^ Up-regulation of miR-21-3p, miR-126-5p, and miR-31-5p and down-regulation of the genes miR-99b and miR-146a were associated with wound healing.^50^ The ratio of M2 macrophages to M1 macrophages was positively correlated with miR-21. In the early stages of inflammation, pri-miR-21 dominates and has pro-inflammatory effects. Conversely, during the repair phase of inflammation, mature miR-21 exerts anti-inflammatory effects and converts macrophages to M2 macrophages, which exhibit low inflammatory levels and an immunosuppressed state, leading to persistent inflammation.^51^^,^^52^ Previous studies have shown that M2-ABs can complete phenotypic reprogramming from the M1 phenotype to the M2 phenotype by targeting M1-mediated delivery of miR-21a-5p.^53^ Similarly, we also found that miR-21a-5p expression was high according to transcriptome sequencing, leading us to hypothesize that miR-21a-5p could also drive Mϕ reprogramming to M2-Mϕ. We found that miR-21a-5p is a key molecule for wound healing and that its reduced expression may be one of the pathogenic molecular mechanisms of impaired M0 to M2 conversion; inhibition of miR-21a-5p expression in MSC apoptotic vesicles silences the target gene CCL-1 and inhibits M0 to M2 conversion. In this study, we validated that MSC-ABs deliver miR-21a-5p to promote the conversion of M0 to M2 macrophages, which in turn exerts anti-inflammatory and pro-angiogenic effects on M2 macrophages, providing theoretical support for an in-depth study of the pathological mechanisms of wound healing.

In this study, we isolated and characterized BMSC-derived ABs and explored their regulatory mechanisms in macrophage programming, and we selected mouse trauma as a disease model. We found that the delivery of miR-21a-5p to the ABs of BMSC-programmed macrophages to M2 macrophages, which target the CCL-1 gene, promoted angiogenesis by secreting the anti-inflammatory cytokines IL-4, IL-10, CCL-1, and TGF-β and the angiogenesis-related factors VEGF and vWF to alter the local inflammatory environment. We found that BMSC treatment effectively promoted wound healing and attenuated the development of early wound inflammation. After the dorsal wounds of mice were covered with PCL fiber scaffolds loaded with apoptotic vesicles from BMSCs, the ABs from the BMSCs significantly accelerated the time to wound healing and promoted the formation of blood vessels, indicating that the use of PCL fiber scaffolds can act synergistically with the ABs. Although this study suggested that ABs from BMSCs are effective at preventing delayed wound healing in mice, the mechanisms by which these improvements in wound healing occur have not been fully determined. Therefore, further study of the additional molecular composition and mechanisms by which BMSCs act as ABs is necessary.

## Conclusions

This study demonstrates for the first time that apoptotic vesicles derived from bone marrow MSCs can induce macrophage M2 polarization and promote skin wound healing by targeting the CCL-1 gene through the presence of mmu-miR-21a-5p. Through electrostatic spinning technology, we prepared PCL composite fiber materials to construct MSC-AB-released carrier scaffolds and targeted the delivery of miR-21a-5p through local slow-release MSC-ABs to drive macrophage M0 to M2 programming to exert its dual effect of inflammation regulation and angiogenesis and then synergistically promote wound healing. In this study, stem cell-derived apoptotic vesicles, cell signaling required for macrophage programming, and PCL scaffolds were used to investigate the immunopathogenic mechanism of wound healing and new therapeutic targets, providing a promising therapeutic strategy and an experimental basis and theoretical rationale for various diseases associated with an imbalance of pro- and anti-inflammatory immune responses.

## Ethics approval

The study was approved by the Animal Ethics Committee of Chongqing Medical University (No. IACU-CQMU-2023-0037). All animal experiments were approved by the Animal Ethics Committee of Chongqing Medical University (No. IACU-CQMU-2023-0037). All authors consent to the publication of this study.

## Funding

This work was supported by the 10.13039/501100001809National Natural Science Foundation of China (No. 82072443, 82372425), the Chongqing Outstanding Project of Overseas Chinese Entrepreneurship and Innovation Support Program (China) (No. CX2022032), the Articular Cartilage Tissue 10.13039/100000084Engineering and Regenerative Medicine Team, 10.13039/501100004374Chongqing Medical University (No. W0080), the General Project of Natural Science Foundation of Chongqing, China (No. CSTB2023NSCQ-MSX0166), the “Tomorrow Cup” Teacher‒Student Cocreation Teaching and Research Innovation Project of International Medical College of Chongqing Medical University (No. KY20220204), the Chengdu Medical Research Project (Sichuan, China) (No. 2023191), the China Postdoctoral Science Foundation (No. 2022M710557), the Natural Science Foundation of Chongqing, China (No. CSTB2023NSCQ-BHX0011), and the Young Excellent Science and Technology Talent Project of the First Affiliated Hospital of Chongqing Medical University (No. ZYRC2022-05).

## Author contributions

Ning Hu, Leilei Qin, and Yonghua Yuan conceived the manuscript. Xudong Su wrote the first draft. Jianye Yang and Xudong Su revised the first draft. Leilei Qin, Xudong Su, and Zhenghao Xu performed the experiments. Wenge He, Li Chen, Shuhao Yang, Li Wei, and Chen Zhao provided grouping suggestions. Ning Hu provided language and grammar modifications. All authors read and approved the final manuscript.

## Data availability

The datasets used and/or analyzed during the current study are available from the corresponding author upon reasonable request.

## Conflict of interests

The authors confirm that there is no conflict of interests.

## Appendix A Supplementary data

Figure S1 Polycaprolactone-loaded bone mesenchymal stem cell-derived apoptotic bodies (PCL-BMSC-ABs) drive the reprogramming of macrophages (Mϕs) to M2-Mϕs. **(A)** The binding of miR-21a-5p to the target gene CCL-1 in RAW 264.7 cells validated by dual luciferase assay. **(B)** Flow cytometry comparison of CD206-positive M1 Mϕs and Mϕs incubated with 50 μg/mL PCL-BMSC-ABs for different periods. **(C)***In vitro* levels of tumor necrosis factor (TNF)-α in reprogrammed PCL-loaded M2-Mϕs (RM2). **(D)***In vitro* levels of TNF-β in reprogrammed PCL-loaded M2-Mϕs (RM2). **(E)***In vitro* levels of von Willebrand factor (vWF) in RM2. **(F)***In vitro* levels of vascular endothelial growth factor (VEGF) in RM2. **(G)** Real-time imaging of Cy7-N-hydroxysuccinimide (NHS)-labeled ABs. n = 3; ∗∗∗P < 0.001, ∗∗P < 0.01, ∗P < 0.05. Figure S1
