# Supplementary figures and images for: Fibrous scaffolds loaded with BMSC-derived apoptotic vesicles promote wound healing by inducing macrophage polarization
Source: Genes Dis. 2024 Aug 9;12(2):101388. doi: 10.1016/j.gendis.2024.101388 (PMC11697094; doi:10.1016/j.gendis.2024.101388)

**A**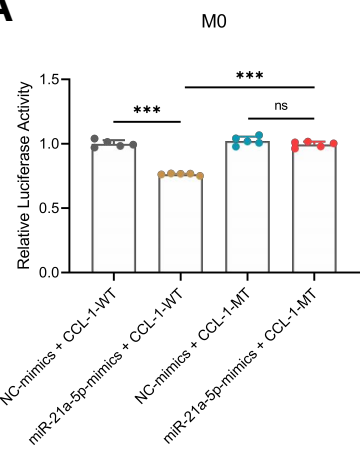**B**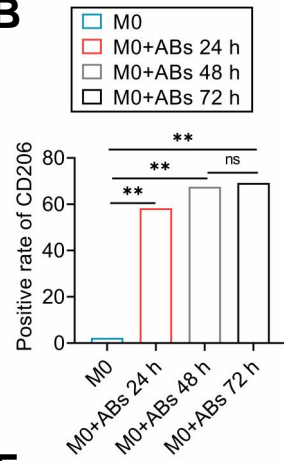**C**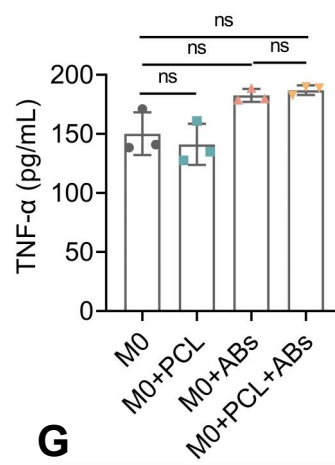**D**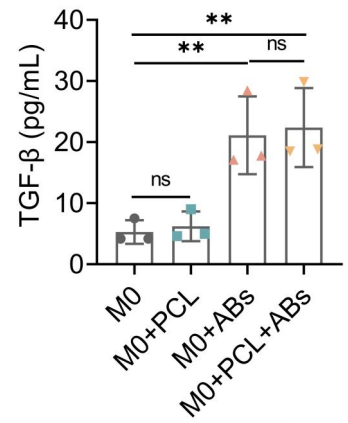**E**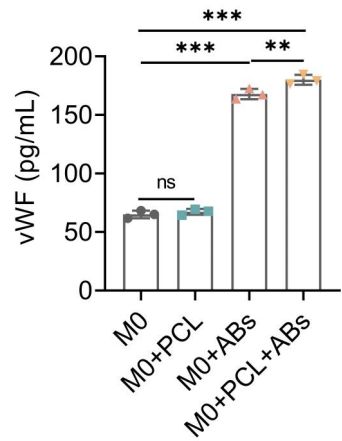**F**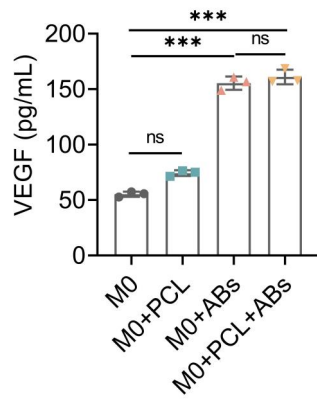**G**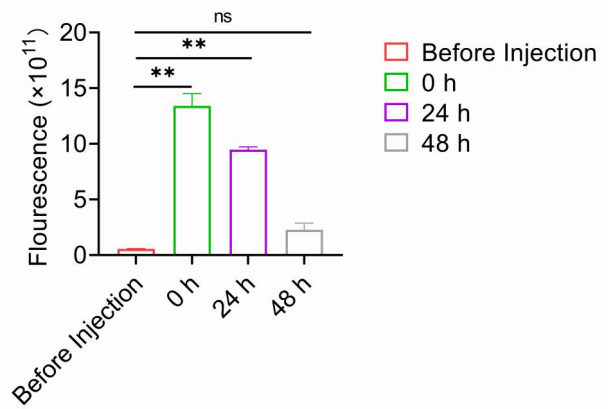

Supplement: Figure S1 — Polycaprolactone-loaded bone mesenchymal stem cell-derived apoptotic bodies (PCL-BMSC-ABs) drive the reprogramming of macrophages (Mϕs) to M2-Mϕs. (A) The binding of miR-21a-5p to the target gene CCL-1 in RAW 264.7 cells validated by dual luciferase assay. (B) Flow cytometry comparison of CD206-positive M1 Mϕs and Mϕs incubated with 50 μg/mL PCL-BMSC-ABs for different periods. (C)In vitro levels of tumor necrosis factor (TNF)-α in reprogrammed PCL-loaded M2-Mϕs (RM2). (D)In vitro levels of TNF-β in reprogrammed PCL-loaded M2-Mϕs (RM2). (E)In vitro levels of von Willebrand factor (vWF) in RM2. (F)In vitro levels of vascular endothelial growth factor (VEGF) in RM2. (G) Real-time imaging of Cy7-N-hydroxysuccinimide (NHS)-labeled ABs. n = 3; ∗∗∗P < 0.001, ∗∗P < 0.01, ∗P < 0.05. [file mmc1.pdf]
